# Correction: Biodegradation mechanism of butralin by a newly isolated *Bacillus velezensis* FC02

**DOI:** 10.1039/d5ra90119g

**Published:** 2025-10-23

**Authors:** Feifei Tan, Wenzheng Ling, Hao Zong, Chuantao Xu, Jiamin Yu, Haichao Cao, Dan Chen, Chao Feng

**Affiliations:** a Key Laboratory of Tobacco Pest Monitoring Controlling & Integrated Management, Tobacco Research Institute of Chinese Academy of Agricultural Sciences Qingdao 266101 China chendan@caas.cn fengchao@caas.cn caohaichao@caas.cn; b Shandong Linyi Tobacco Co., Ltd Linyi 276000 Shandong China; c Sichuan Tobacco Co., Ltd Chengdu 610000 Sichuan China

## Abstract

Correction for ‘Biodegradation mechanism of butralin by a newly isolated *Bacillus velezensis* FC02’ by Feifei Tan *et al.*, *RSC Adv.*, 2025, **15**, 37461–37473, https://doi.org/10.1039/D5RA02138C.

The authors regret the omission of an Acknowledgments section from the original article. The correct acknowledgment is as shown below.

## Acknowledgements

The work was supported by the Qingdao Technology for Public Well-being Demonstration Project (25-1-5-xdny-6-nsh) and Major technology projects [110202201029 (LS-13)].

The Royal Society of Chemistry apologises for these errors and any consequent inconvenience to authors and readers.

